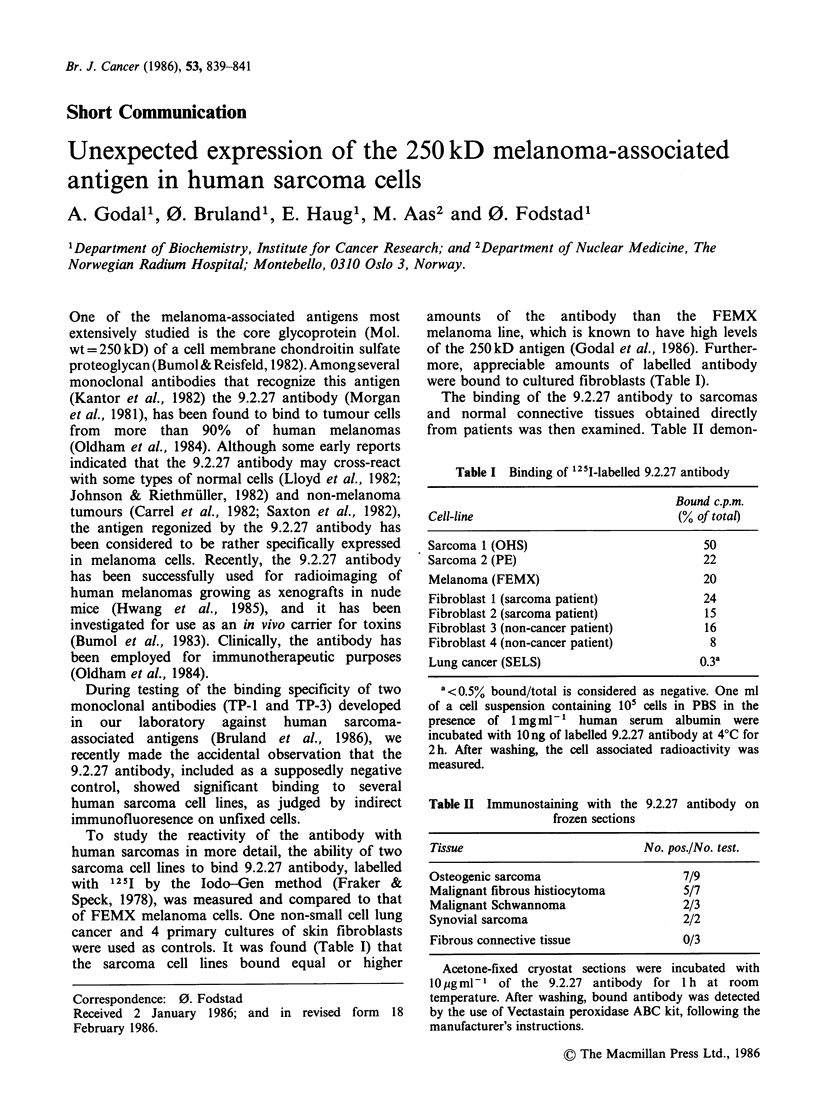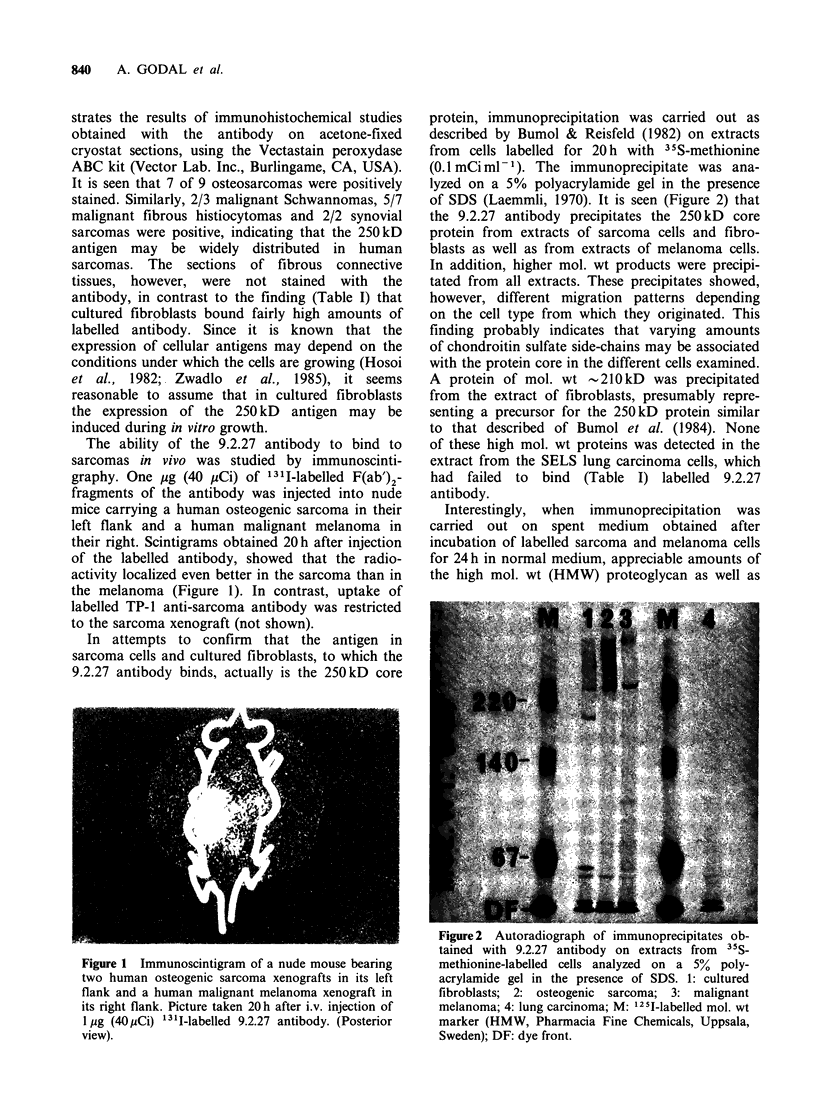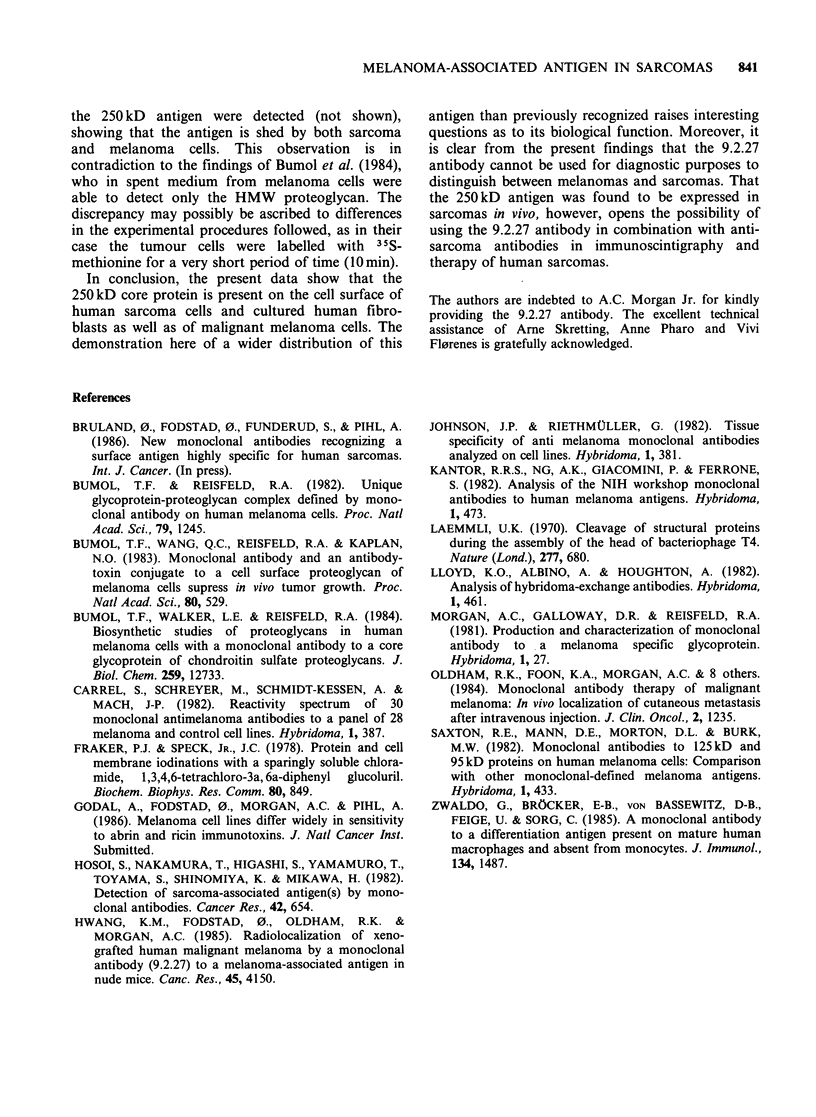# Unexpected expression of the 250 kD melanoma-associated antigen in human sarcoma cells.

**DOI:** 10.1038/bjc.1986.142

**Published:** 1986-06

**Authors:** A. Godal, O. Bruland, E. Haug, M. Aas, O. Fodstad

## Abstract

**Images:**


Br. J. Cancer (1986), **53**, 839–841

**Short Communication**

### Unexpected expression of the 250 kD melanoma-associated antigen in human sarcoma cells

A. Godal[1], Ø. Bruland[1], E. Haug[1], M. Aas[2] and Ø. Fodstad[1]

[1]*Department of Biochemistry, Institute for Cancer Research; and* [2]*Department of Nuclear Medicine, The Norwegian Radium Hospital; Montebello, 0310 Oslo 3, Norway.*

One of the melanoma-associated antigens most extensively studied is the core glycoprotein (Mol. wt = 250 kD) of a cell membrane chondroitin sulfate proteoglycan (Bumol & Reisfeld, 1982). Among several monoclonal antibodies that recognize this antigen (Kantor *et al.*, 1982) the 9.2.27 antibody (Morgan *et al.*, 1981), has been found to bind to tumour cells from more than 90% of human melanomas (Oldham *et al.*, 1984). Although some early reports indicated that the 9.2.27 antibody may cross-react with some types of normal cells (Lloyd *et al.*, 1982; Johnson & Riethmüller, 1982) and non-melanoma tumours (Carrel *et al.*, 1982; Saxton *et al.*, 1982), the antigen regonized by the 9.2.27 antibody has been considered to be rather specifically expressed in melanoma cells. Recently, the 9.2.27 antibody has been successfully used for radioimaging of human melanomas growing as xenografts in nude mice (Hwang *et al.*, 1985), and it has been investigated for use as an *in vivo* carrier for toxins (Bumol *et al.*, 1983). Clinically, the antibody has been employed for immunotherapeutic purposes (Oldham *et al.*, 1984).

During testing of the binding specificity of two monoclonal antibodies (TP-1 and TP-3) developed in our laboratory against human sarcoma-associated antigens (Bruland *et al.*, 1986), we recently made the accidental observation that the 9.2.27 antibody, included as a supposedly negative control, showed significant binding to several human sarcoma cell lines, as judged by indirect immunofluoresence on unfixed cells.

To study the reactivity of the antibody with human sarcomas in more detail, the ability of two sarcoma cell lines to bind 9.2.27 antibody, labelled with $^{125}$I by the Iodo-Gen method (Fraker & Speck, 1978), was measured and compared to that of FEMX melanoma cells. One non-small cell lung cancer and 4 primary cultures of skin fibroblasts were used as controls. It was found (Table I) that the sarcoma cell lines bound equal or higher amounts of the antibody than the FEMX melanoma line, which is known to have high levels of the 250 kD antigen (Godal *et al.*, 1986). Furthermore, appreciable amounts of labelled antibody were bound to cultured fibroblasts (Table I).

The binding of the 9.2.27 antibody to sarcomas and normal connective tissues obtained directly from patients was then examined. Table II demon-

**Table I** Binding of $^{125}$I-labelled 9.2.27 antibody

| *Cell-line* | *Bound c.p.m. (% of total)* |
|---|---|
| Sarcoma 1 (OHS) | 50 |
| Sarcoma 2 (PE) | 22 |
| Melanoma (FEMX) | 20 |
| Fibroblast 1 (sarcoma patient) | 24 |
| Fibroblast 2 (sarcoma patient) | 15 |
| Fibroblast 3 (non-cancer patient) | 16 |
| Fibroblast 4 (non-cancer patient) | 8 |
| Lung cancer (SELS) | 0.3[a] |

[a]<0.5% bound/total is considered as negative. One ml of a cell suspension containing $10^5$ cells in PBS in the presence of $1\,mg\,ml^{-1}$ human serum albumin were incubated with 10 ng of labelled 9.2.27 antibody at 4°C for 2 h. After washing, the cell associated radioactivity was measured.

**Table II** Immunostaining with the 9.2.27 antibody on frozen sections

| *Tissue* | *No. pos./No. test.* |
|---|---|
| Osteogenic sarcoma | 7/9 |
| Malignant fibrous histiocytoma | 5/7 |
| Malignant Schwannoma | 2/3 |
| Synovial sarcoma | 2/2 |
| Fibrous connective tissue | 0/3 |

Acetone-fixed cryostat sections were incubated with $10\,\mu g\,ml^{-1}$ of the 9.2.27 antibody for 1 h at room temperature. After washing, bound antibody was detected by the use of Vectastain peroxidase ABC kit, following the manufacturer's instructions.

Correspondence: Ø. Fodstad
Received 2 January 1986; and in revised form 18 February 1986.

© The Macmillan Press Ltd., 1986

strates the results of immunohistochemical studies obtained with the antibody on acetone-fixed cryostat sections, using the Vectastain peroxydase ABC kit (Vector Lab. Inc., Burlingame, CA, USA). It is seen that 7 of 9 osteosarcomas were positively stained. Similarly, 2/3 malignant Schwannomas, 5/7 malignant fibrous histiocytomas and 2/2 synovial sarcomas were positive, indicating that the 250 kD antigen may be widely distributed in human sarcomas. The sections of fibrous connective tissues, however, were not stained with the antibody, in contrast to the finding (Table I) that cultured fibroblasts bound fairly high amounts of labelled antibody. Since it is known that the expression of cellular antigens may depend on the conditions under which the cells are growing (Hosoi *et al.*, 1982; Zwadlo *et al.*, 1985), it seems reasonable to assume that in cultured fibroblasts the expression of the 250 kD antigen may be induced during *in vitro* growth.

The ability of the 9.2.27 antibody to bind to sarcomas *in vivo* was studied by immunoscintigraphy. One $\mu g$ (40 $\mu Ci$) of $^{131}I$-labelled $F(ab')_2$-fragments of the antibody was injected into nude mice carrying a human osteogenic sarcoma in their left flank and a human malignant melanoma in their right. Scintigrams obtained 20 h after injection of the labelled antibody, showed that the radioactivity localized even better in the sarcoma than in the melanoma (Figure 1). In contrast, uptake of labelled TP-1 anti-sarcoma antibody was restricted to the sarcoma xenograft (not shown).

In attempts to confirm that the antigen in sarcoma cells and cultured fibroblasts, to which the 9.2.27 antibody binds, actually is the 250 kD core protein, immunoprecipitation was carried out as described by Bumol & Reisfeld (1982) on extracts from cells labelled for 20 h with $^{35}S$-methionine ($0.1\,mCi\,ml^{-1}$). The immunoprecipitate was analyzed on a 5% polyacrylamide gel in the presence of SDS (Laemmli, 1970). It is seen (Figure 2) that the 9.2.27 antibody precipitates the 250 kD core protein from extracts of sarcoma cells and fibroblasts as well as from extracts of melanoma cells. In addition, higher mol. wt products were precipitated from all extracts. These precipitates showed, however, different migration patterns depending on the cell type from which they originated. This finding probably indicates that varying amounts of chondroitin sulfate side-chains may be associated with the protein core in the different cells examined. A protein of mol. wt ~210 kD was precipitated from the extract of fibroblasts, presumably representing a precursor for the 250 kD protein similar to that described of Bumol *et al.* (1984). None of these high mol. wt proteins was detected in the extract from the SELS lung carcinoma cells, which had failed to bind (Table I) labelled 9.2.27 antibody.

Interestingly, when immunoprecipitation was carried out on spent medium obtained after incubation of labelled sarcoma and melanoma cells for 24 h in normal medium, appreciable amounts of the high mol. wt (HMW) proteoglycan as well as

**Figure 1** Immunoscintigram of a nude mouse bearing two human osteogenic sarcoma xenografts in its left flank and a human malignant melanoma xenograft in its right flank. Picture taken 20 h after i.v. injection of 1 $\mu g$ (40 $\mu Ci$) $^{131}I$-labelled 9.2.27 antibody. (Posterior view).

**Figure 2** Autoradiograph of immunoprecipitates obtained with 9.2.27 antibody on extracts from $^{35}S$-methionine-labelled cells analyzed on a 5% polyacrylamide gel in the presence of SDS. 1: cultured fibroblasts; 2: osteogenic sarcoma; 3: malignant melanoma; 4: lung carcinoma; M: $^{125}I$-labelled mol. wt marker (HMW, Pharmacia Fine Chemicals, Uppsala, Sweden); DF: dye front.

the 250 kD antigen were detected (not shown), showing that the antigen is shed by both sarcoma and melanoma cells. This observation is in contradiction to the findings of Bumol *et al.* (1984), who in spent medium from melanoma cells were able to detect only the HMW proteoglycan. The discrepancy may possibly be ascribed to differences in the experimental procedures followed, as in their case the tumour cells were labelled with $^{35}$S-methionine for a very short period of time (10 min).

In conclusion, the present data show that the 250 kD core protein is present on the cell surface of human sarcoma cells and cultured human fibroblasts as well as of malignant melanoma cells. The demonstration here of a wider distribution of this antigen than previously recognized raises interesting questions as to its biological function. Moreover, it is clear from the present findings that the 9.2.27 antibody cannot be used for diagnostic purposes to distinguish between melanomas and sarcomas. That the 250 kD antigen was found to be expressed in sarcomas *in vivo,* however, opens the possibility of using the 9.2.27 antibody in combination with anti-sarcoma antibodies in immunoscintigraphy and therapy of human sarcomas.

The authors are indebted to A.C. Morgan Jr. for kindly providing the 9.2.27 antibody. The excellent technical assistance of Arne Skretting, Anne Pharo and Vivi Flørenes is gratefully acknowledged.

### References

BRULAND, Ø., FODSTAD, Ø., FUNDERUD, S., & PIHL, A. (1986). New monoclonal antibodies recognizing a surface antigen highly specific for human sarcomas. *Int. J. Cancer.* (In press).

BUMOL, T.F. & REISFELD, R.A. (1982). Unique glycoprotein-proteoglycan complex defined by monoclonal antibody on human melanoma cells. *Proc. Natl Acad. Sci.,* **79,** 1245.

BUMOL, T.F., WANG, Q.C., REISFELD, R.A. & KAPLAN, N.O. (1983). Monoclonal antibody and an antibody-toxin conjugate to a cell surface proteoglycan of melanoma cells supress *in vivo* tumor growth. *Proc. Natl Acad. Sci.,* **80,** 529.

BUMOL, T.F., WALKER, L.E. & REISFELD, R.A. (1984). Biosynthetic studies of proteoglycans in human melanoma cells with a monoclonal antibody to a core glycoprotein of chondroitin sulfate proteoglycans. *J. Biol. Chem.* **259,** 12733.

CARREL, S., SCHREYER, M., SCHMIDT-KESSEN, A. & MACH, J-P. (1982). Reactivity spectrum of 30 monoclonal antimelanoma antibodies to a panel of 28 melanoma and control cell lines. *Hybridoma,* **1,** 387.

FRAKER, P.J. & SPECK, JR., J.C. (1978). Protein and cell membrane iodinations with a sparingly soluble chloramide, 1,3,4,6-tetrachloro-3a, 6a-diphenyl glucoluril. *Biochem. Biophys. Res. Comm.* **80,** 849.

GODAL, A., FODSTAD, Ø., MORGAN, A.C. & PIHL, A. (1986). Melanoma cell lines differ widely in sensitivity to abrin and ricin immunotoxins. *J. Natl Cancer Inst.* Submitted.

HOSOI, S., NAKAMURA, T., HIGASHI, S., YAMAMURO, T., TOYAMA, S., SHINOMIYA, K. & MIKAWA, H. (1982). Detection of sarcoma-associated antigen(s) by monoclonal antibodies. *Cancer Res.,* **42,** 654.

HWANG, K.M., FODSTAD, Ø., OLDHAM, R.K. & MORGAN, A.C. (1985). Radiolocalization of xenografted human malignant melanoma by a monoclonal antibody (9.2.27) to a melanoma-associated antigen in nude mice. *Canc. Res.,* **45,** 4150.

JOHNSON, J.P. & RIETHMÜLLER, G. (1982). Tissue specificity of anti melanoma monoclonal antibodies analyzed on cell lines. *Hybridoma,* **1,** 381.

KANTOR, R.R.S., NG, A.K., GIACOMINI, P. & FERRONE, S. (1982). Analysis of the NIH workshop monoclonal antibodies to human melanoma antigens. *Hybridoma,* **1,** 473.

LAEMMLI, U.K. (1970). Cleavage of structural proteins during the assembly of the head of bacteriophage T4. *Nature* (*Lond.*), **277,** 680.

LLOYD, K.O., ALBINO, A. & HOUGHTON, A. (1982). Analysis of hybridoma-exchange antibodies. *Hybridoma,* **1,** 461.

MORGAN, A.C., GALLOWAY, D.R. & REISFELD, R.A. (1981). Production and characterization of monoclonal antibody to a melanoma specific glycoprotein. *Hybridoma,* **1,** 27.

OLDHAM, R.K., FOON, K.A., MORGAN, A.C. & 8 others. (1984). Monoclonal antibody therapy of malignant melanoma: *In vivo* localization of cutaneous metastasis after intravenous injection. *J. Clin. Oncol.,* **2,** 1235.

SAXTON, R.E., MANN, D.E., MORTON, D.L. & BURK, M.W. (1982). Monoclonal antibodies to 125 kD and 95 kD proteins on human melanoma cells: Comparison with other monoclonal-defined melanoma antigens. *Hybridoma,* **1,** 433.

ZWALDO, G., BRÖCKER, E-B., VON BASSEWITZ, D-B., FEIGE, U. & SORG, C. (1985). A monoclonal antibody to a differentiation antigen present on mature human macrophages and absent from monocytes. *J. Immunol.,* **134,** 1487.